# Surgical Repair of Double-Outlet Right Ventricle With Doubly Committed Juxtaarterial Ventricular Septal Defect: The Role of Virtual Reality

**DOI:** 10.1016/j.atssr.2025.07.016

**Published:** 2025-08-20

**Authors:** Fumiya Yoneyama, Jonathan Awori, Shagun Sachdeva, Kaimal A. Jayakumar, Javier Brenes, Jeffery S. Heinle

**Affiliations:** 1 Department of Congenital Heart Surgery, Texas Children’s Hospital, Baylor College of Medicine, Houston, Texas; 2 Department of Pediatric Cardiology, Texas Children’s Hospital, Baylor College of Medicine, Houston, Texas

## Abstract

Double-outlet right ventricle (DORV) with a doubly committed juxtaarterial ventricular septal defect (VSD) presents significant surgical challenges. We report a neonate with DORV, a hypoplastic aortic arch, and a patent ductus arteriosus. Virtual reality (VR) imaging provided detailed visualization, confirming the VSD’s position beneath both great arteries. Intraventricular rerouting was unfeasible given the patient’s small systemic structures and tricuspid valve chordae. The patient underwent successful VSD closure through a transarterial approach, arterial switch operation, and arch repair. This case highlights the utility of VR-based planning in optimizing surgical strategy for complex congenital heart defects.

Double-outlet right ventricle (DORV) with a doubly committed juxtaarterial ventricular septal defect (VSD) is a rare congenital anomaly presenting complex anatomic challenges.^1^^,^^2^ In this variant, both great arteries arise from the right ventricle (RV), with the VSD located beneath the semilunar valves and often associated with a fibrous outlet septum. These anatomic relationships complicate surgical planning and ventricular septation.

Surgical management of DORV with a doubly committed juxtaarterial VSD depends on factors such as great artery relationships, obstructive muscle bundles, and the feasibility of creating an unobstructed left ventricular outflow tract. Two main approaches include intraventricular rerouting (left ventricle [LV] to aorta) and an arterial switch operation (ASO) with VSD closure.^1^^,^^3^ ASO is often preferred in subpulmonary VSDs, whereas rerouting may be limited by subaortic stenosis or obstructive tricuspid chordae.^3^^,^^4^ Accurate preoperative imaging is essential in selecting the optimal strategy. Recently, virtual reality (VR) has become a valuable tool in congenital cardiac surgery, by offering interactive, patient-specific 3-dimensional visualization of anatomy.^5^^,^^6^ VR has been shown to improve surgical planning, guide decision making, and align closely with intraoperative findings.^7^

Here we report the case of a neonate with DORV, a doubly committed juxtaarterial VSD, a hypoplastic aortic arch, and a patent ductus arteriosus (PDA). Preoperative VR modeling played a key role in defining anatomic relationships, confirming surgical feasibility, and guiding the successful approach involving ASO, VSD closure, and arch repair.

A neonate weighing 3.5 kg was born by an uncomplicated delivery and was given a diagnosis of DORV, a large and doubly committed juxtaarterial VSD, a hypoplastic aortic arch, and a PDA. Echocardiography revealed a large, doubly committed VSD with a minimal outlet septum between the aortic and pulmonary valves, a widely patent PDA, and a patent foramen ovale. The aortic valve annulus size was 6.3 mm, whereas the pulmonary valve was 9.3 mm (Figure 1A). Computed tomography demonstrated that the aorta and pulmonary artery gave rise from the RV in a side-by-side relationship, with a hypoplastic ascending and transverse arch with a small isthmus (Figure 1B).

**Figure 1:**
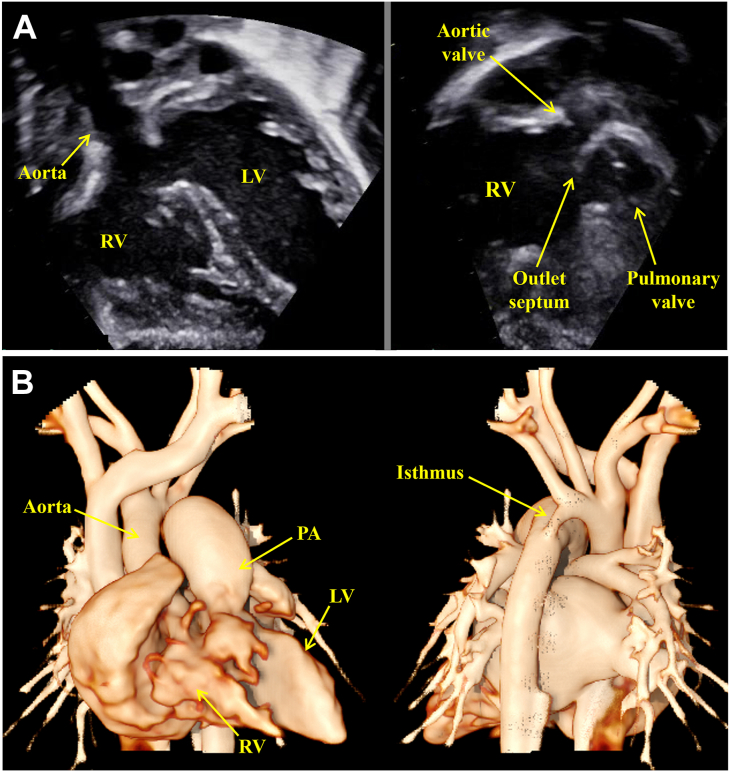
(A) Preoperative transthoracic echocardiography. (B) Preoperative 3-dimensional computed tomographic images. (LV, left ventricle; PA, pulmonary artery; RV, right ventricle.)

Given the anatomic complexity of this case, a 3-dimensional VR model was developed using Elucis software (Realize Medical) for preoperative planning. The model confirmed that the VSD was located directly beneath the great arteries, with a minimal outlet septum between the aortic and pulmonary valves, although more committed to the pulmonary valve (Figure 2). There was concern regarding the small structures of the systemic arterial system, including the aortic valve, ascending aorta, and aortic arch, despite the expectation that the aortic arch would be augmented postoperatively. Therefore, VSD closure (LV to neoaorta baffling) combined with an ASO was considered a more favorable approach than intraventricular rerouting (LV to aortic valve). Additionally, the crest of the VSD was clearly visible through the transected aorta and pulmonary artery in the reconstructed VR images, thus making a transarterial approach for doubly committed VSD closure both feasible and technically advantageous.

**Figure 2:**
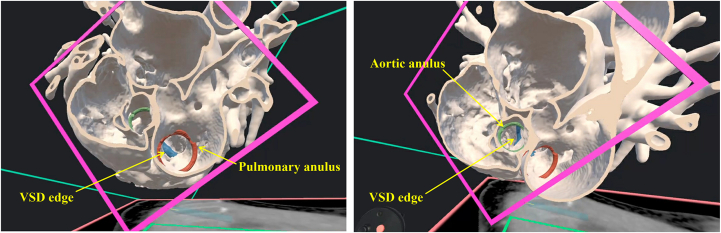
Virtual reality images. The crest of the ventricular septum (blue) from the pulmonary valve (red) as well as the aortic valve (green) can be visualized. (VSD, ventricular septal defect.)

The patient was brought to the operating room at the age of 5 days. After median sternotomy and cardiopulmonary bypass induction, the ascending aorta was cross-clamped, and cardiac arrest was achieved with cardioplegia. The ascending aorta was divided slightly above the sinutubular junction, and antegrade cerebral perfusion was initiated. After obliterating the PDA tissue, the descending aorta was anastomosed directly to the posterior wall of the opened ascending aorta and aortic arch, followed by patch augmentation of the anterior wall of the neoaortic arch. A cross-clamp was placed on the reconstructed ascending aorta to resume full-body perfusion.

A large, doubly committed VSD with a minimal fibrous outlet septum was confirmed (Figure 3). Some chordae from the tricuspid valve were attached to the crest of the ventricular septum, thereby posing a significant challenge and ultimately rendering intracardiac rerouting unfeasible. The VSD was closed with an autologous pericardial patch through the neoaortic annulus for the anterior VSD portion (red line of A in Figure 3), and the posterior potion of the VSD was closed through the neopulmonary annulus (red line of B in Figure 3). The suture for the superior potion of the VSD was anchored to the neopulmonary and neoaortic annulus tissues (blue lines of C and D in Figure 3). The coronary pattern was 1R-2LCx, and the coronary arteries were transferred to the neoaorta. The reconstructed pulmonary arteries were positioned behind the aortic arch. Separation from cardiopulmonary bypass was achieved without difficulty. Transesophageal echocardiography demonstrated no obstruction in the left and right outflow tracts, no significant neoaortic insufficiency, no residual VSD, and good ventricular function. The patient was extubated on postoperative day 3, and was discharged home on postoperative day 12 after an uneventful recovery.

**Figure 3:**
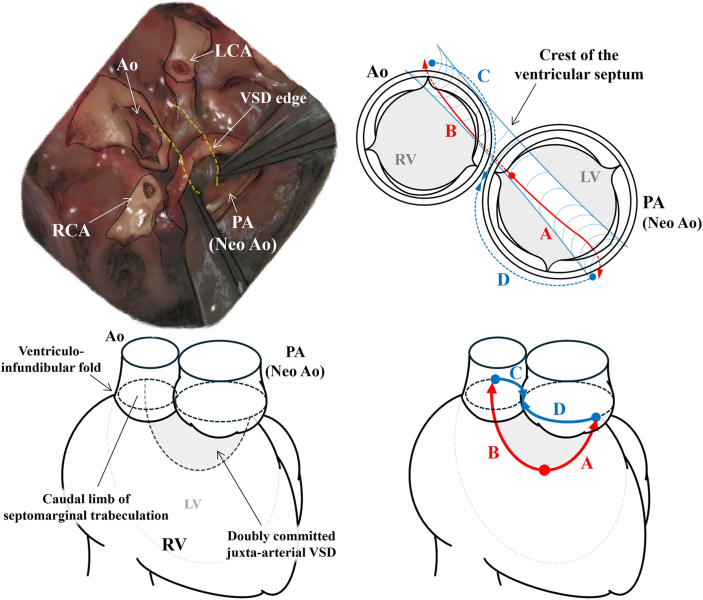
Intraoperative images. The juxtaarterial ventricular septal defect (VSD) was closed using a transarterial approach. Following the indicated arrows, the running sutures were placed sequentially from points A to D. The red arrows (A, B) mark sutures placed along the edge of the VSD, whereas the blue arrows (C, D) indicate sutures placed along the semilunar valve annulus. (Ao, aorta; LCA, left coronary artery; LV, left ventricle; PA, pulmonary artery; RCA, right coronary artery; RV, right ventricle.)

## Comment

DORV with a doubly committed juxtaarterial VSD is a complex congenital heart defect, characterized by both great arteries arising from the RV and the VSD positioned beneath both semilunar valves.^1^^,^^2^ Surgical repair often involves intraventricular rerouting from the LV to the aorta, frequently requiring muscle bundle resection. However, subaortic stenosis and the risk of reoperation remain major concerns.^1^^,^^3^ The morphology of the outlet septum significantly influences both classification and surgical strategy in DORV-associated VSDs.^2^^,^^8^ In patients with a subpulmonary VSD, the ASO has become the preferred approach, with favorable long-term survival.^5^

Given this anatomic complexity, advanced imaging, particularly VR, has become vital for surgical planning. VR and mixed reality tools improve understanding of spatial relationships and have influenced surgical decisions in up to 68% of cases,^5^^,^^6^ with a 95% match between VR planning and intraoperative findings.^7^ In our patient, VR clearly depicted the VSD beneath both semilunar valves with a minimal outlet septum, thus confirming that transarterial closure was feasible. This patient-specific model allowed the team to minimize aortic cross-clamp and circulatory arrest times—critical in the context of arch repair and ASO—demonstrating the value of VR-based planning in complex congenital repairs.
